# Soft Iontronic Diodes: Materials, Mechanisms, and Progress

**DOI:** 10.3390/gels12070656

**Published:** 2026-07-22

**Authors:** Liang Li, Qinchen Meng, Li Wang

**Affiliations:** School of Chemistry and Molecular Engineering, Nanjing Tech University, Nanjing 211816, China; 202321038017@njtech.edu.cn (L.L.); mengqinchen@njtech.edu.cn (Q.M.)

**Keywords:** soft iontronics, diodes, rectification, gels, elastomers, neuromorphic

## Abstract

Soft iontronic devices, which utilize ions as charge carriers and integrate flexibility and stretchability, exhibit diverse carrier species, high biocompatibility, multimodal stimulus responsiveness, and strong resistance to electromagnetic interference. These features make them highly promising for applications in ionic circuits, flexible sensing, implantable systems, and neuromorphic information processing. Among them, soft iontronic diodes have attracted sustained attention over the past two decades as fundamental building blocks of functional circuits. This review systematically summarizes the material types of soft iontronic diodes and their influence on key device performance. It further elucidates the mechanisms underlying ionic rectification and highlights recent advances in logic gate implementation, energy harvesting, flexible sensing, and neuromorphic computing. Finally, we discuss key challenges and future opportunities in this field, aiming to provide design principles and mechanistic insights for the development and application of soft iontronic diodes.

## 1. Introduction

Soft iontronics employs ions as charge carriers in conjunction with soft polymeric networks, including hydrogels, ionogels, and ionic elastomers, to develop ionic devices that exhibit exceptional stretchability, high optical transparency, and excellent biocompatibility [1,2,3,4,5]. Compared with conventional electronics, ions possess unique physicochemical characteristics, including greater chemical diversity, larger mass and slower mobility [6], and the ability to accumulate at interfaces to form ultrahigh-capacitance electric double layers [7,8,9,10]. These distinctive properties enable soft ionic devices to achieve mechanical compatibility and efficient biointerfacing with biological tissues while maintaining stable ionic conductivity under large tensile or torsional deformations [11]. Within iontronic systems, ionic diodes serve as key building blocks for regulating directional ion transport. Their operating principle is analogous to that of conventional electronic diodes that govern unidirectional electron flow: under forward bias, ionic transport is facilitated, whereas under reverse bias, depletion regions form to suppress ionic conduction [12,13,14]. This ionic current rectification behavior effectively overcomes the intrinsic limitation of isotropic ion diffusion and is therefore critical for enabling ionic logic gates, ionic transistors, and biomimetic neuromorphic circuits. Consequently, ionic diodes provide an essential theoretical and device foundation for emerging applications in intelligent artificial skin, implantable neural interfaces, and next-generation human–machine interaction systems [15,16,17,18].

Since the first ionic diode based on micro/nanofabrication technology was reported in 2007, iontronics has gradually evolved from conventional rigid devices toward flexible, integrated, and intelligent systems [19]. Early ionic diodes primarily relied on nanochannels, ion-exchange membranes, and microfluidic architectures to achieve unidirectional ion transport, with research efforts mainly focused on enhancing ion selectivity and ionic current rectification performance. At present, the performance of soft ionic diodes has improved substantially, with rectification ratios increasing from single-digit values in early systems to values exceeding 10^3^ [15]. Some devices are even capable of maintaining stable ionic rectification behavior under complex mechanical deformations [20,21,22]. Moreover, the functionality of soft ionic diodes has expanded beyond simple ion rectification to include flexible sensing, energy conversion, ionic logic operations, ionic transistors, and biomimetic neuromorphic computing, thereby providing new opportunities for the development of next-generation intelligent bioelectronic devices and human–machine interfacing systems [23,24,25,26,27,28,29]. Despite these advances, soft ionic diodes are still in the early stages of development and continue to face several critical challenges. Hydrogel-based systems commonly suffer from solvent evaporation, freezing at low temperatures, and insufficient long-term stability. Although ionogels generally exhibit superior environmental stability, achieving an optimal balance among mechanical robustness, ionic conductivity, and ion transport efficiency remains difficult [30,31,32,33,34,35]. In addition, the trade-off between rapid response speed and high rectification performance has not yet been fully understood. Other unresolved issues, including interfacial stability, large-scale device integration, and long-term cycling reliability, also require further investigation. Consequently, the rational design of materials, interfacial engineering strategies, and device architectures to simultaneously achieve high stability, strong rectification performance, and multifunctional integration has become a central research focus in the field of soft ionic diodes [30,36,37].

**Figure 1:**
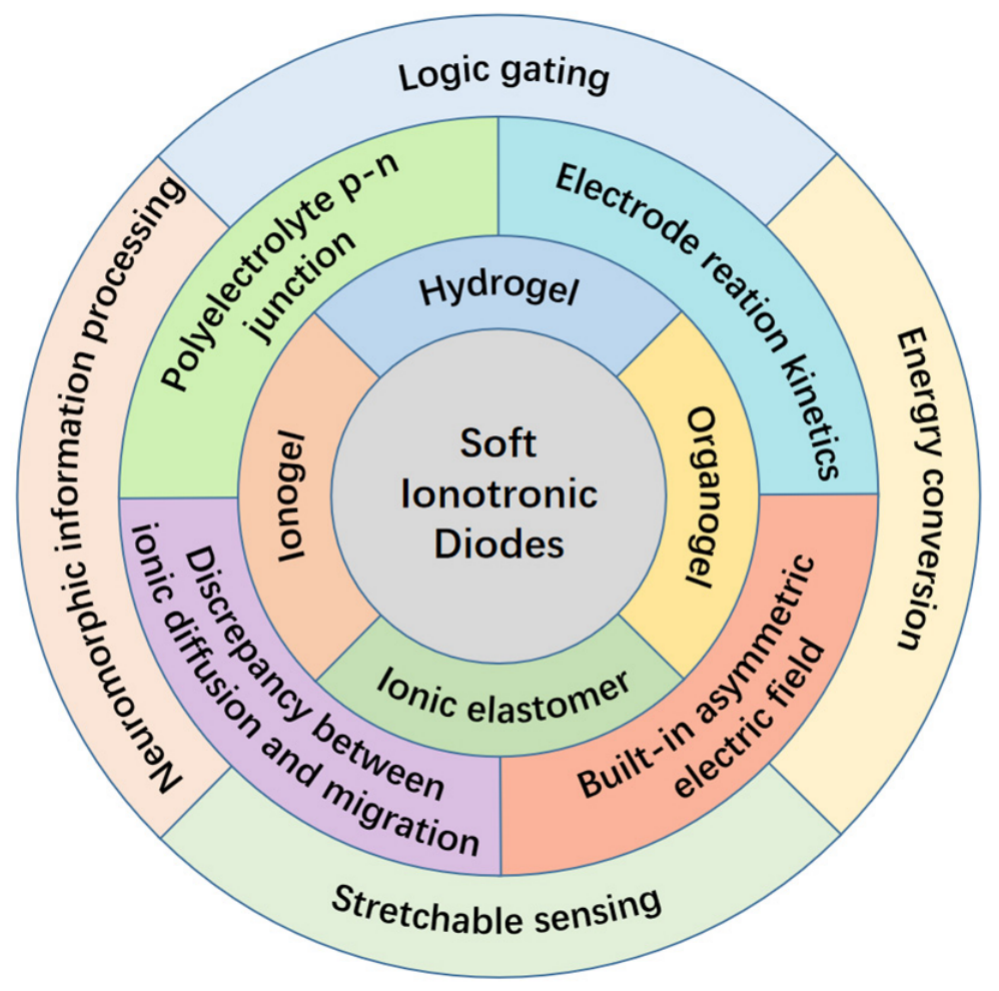
Schematic outline of this review.

Although several excellent review articles have recently summarized the progress of iontronics, ionic devices, and soft iontronic systems, most of them mainly focus on specific material systems, device types, or application fields [2,6,38]. A comprehensive review that systematically correlates the relationships among material systems, rectification mechanisms, device performance, and emerging applications of soft iontronic diodes is still lacking. This review aims to summarize recent advances and future development opportunities in the field of soft iontronic diodes from the perspectives of material systems, rectification mechanisms, and application scenarios. The present article provides a concise discussion centered specifically on ionic diodes, while broader applications, such as neuromorphic iontronic systems, can be found in our previous work. First, the structural characteristics and corresponding performance of different material platforms, including hydrogels, organogels, ionogels, and ionic elastomers, are systematically compared. Subsequently, the underlying ionic rectification mechanisms are analyzed in depth, with particular emphasis on the advantages and limitations associated with different rectification strategies. The review then highlights recent progress in the application of ionic diodes in emerging areas such as ionic logic gates, energy conversion, brain-inspired information processing, and biosensing. Finally, a forward-looking perspective on the challenges and opportunities in this rapidly emerging field is presented, with particular focus on the future development of soft ionic diodes (Figure 1).

## 2. Material Types

Material selection dictates the performance of ionic diodes. Depending on the medium type, current mainstream systems can be categorized into four classes: hydrogels, organogels, ionogels, and ionic elastomers. Each category exhibits distinct advantages and trade-offs in ionic conductivity, mechanical flexibility, environmental stability, and biocompatibility, making them suitable for disparate application scenarios. To provide a clear reference, we summarize the variations in their ionic rectification performance and environmental stability in Table 1.

Although different material platforms provide unique advantages, no single material system can simultaneously maximize ionic conductivity, mechanical robustness, environmental stability, rectification ratio, and response speed. Instead, soft iontronic diode materials occupy different regions within a multidimensional performance space. Hydrogels possess the highest potential for both ionic conductivity and rectification ratios, making them well-suited for short-term or encapsulated applications that demand high performance but tolerate lower environmental stability. Organogels balance high rectification ratios with wide-temperature stability, rendering them suitable for multifunctional integration. Ionogels feature outstanding advantages in environmental stability and operating temperature windows, rendering them ideal for open-environment and extreme-temperature scenarios. Ionic elastomers fundamentally eliminate the issue of solvent evaporation, making them highly applicable for long-term implantable or wearable devices, though their ionic conductivity requires further improvement.

### 2.1. Hydrogel

**Figure 2:**
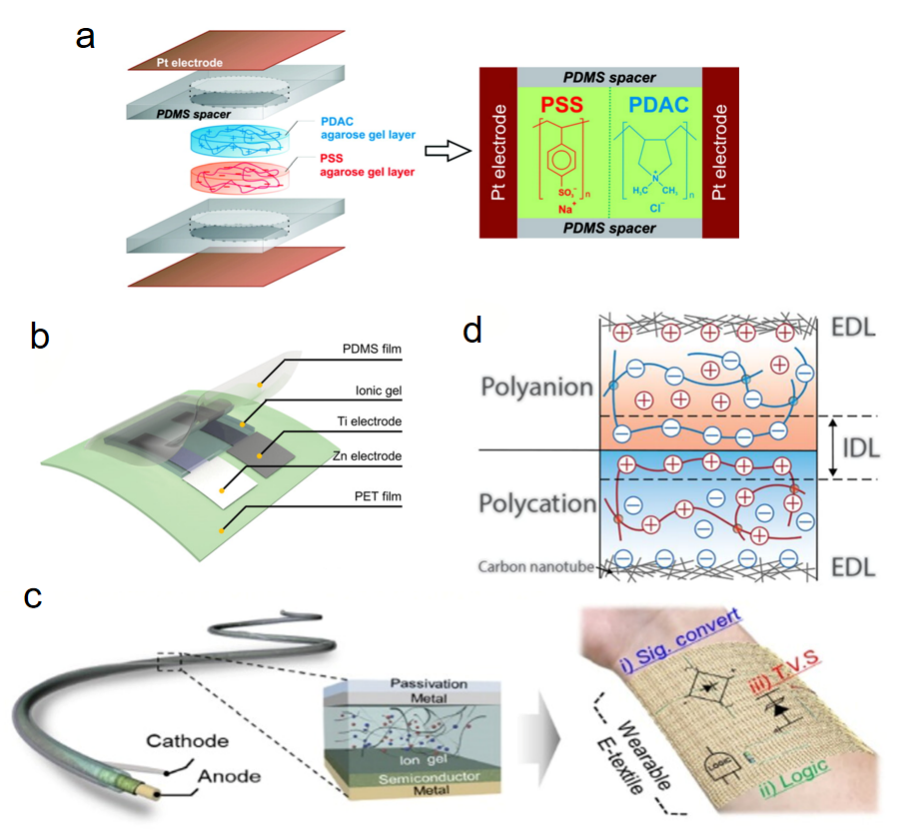
Representative ionic diode devices based on four materials. (**a**,**b**) A hydrogel device [12,42]. (**c**) An ionogel device [53]. (**d**) An ionic elastomer device [31].

Hydrogels are three-dimensional network materials formed by hydrophilic polymer networks that absorb a substantial amount of water. In the field of ionic diodes, hydrogels represent the earliest investigated and utilized matrix materials. As a polar solvent, water effectively dissociates electrolyte salts (such as LiCl, NaCl, and KCl), yielding free ions with high mobility [39,40,41]. In ionic diodes, hydrogels are typically doped with polyelectrolytes to introduce fixed charges, thereby forming a p-n junction structure. Cayre et al. utilized an agarose matrix doped with PSS (polyanion) and PDAC (polycation) to achieve the first rectified hydrogel ionic diode, which exhibited a rectification ratio of approximately 40 (Figure 2a) [12]. Subsequently, Zhang et al. fabricated a biodegradable ionic diode using microfibrillated cellulose and agarose, achieving a rectification ratio of around 15 [42].

In later stages, continuous experimental efforts were dedicated to enhancing the rectification ratio. Guo et al. constructed an ionic diode based on a polyacrylamide (PAAm) hydrogel and an ethylene glycol/water binary solvent by exploiting the massive discrepancy in hydrogen evolution overpotential between Zn and Ti electrodes (Figure 2b). This device achieved a rectification ratio as high as 1201 in a pure water solvent. It is the highest value reported to date while simultaneously demonstrating excellent flexibility and a broad temperature tolerance window (–20 °C to 100 °C) [43]. Wang et al. developed a stretchable ionic diode based on a PAAm hydrogel and PSS/PDAC, which maintained a rectification ratio of 5.6 under a stretch of λ = 4.0, and successfully constructed OR and AND ionic logic gates [14]. Lee et al. reinforced polysaccharides (hyaluronic acid and chitosan) to fabricate stretchable ionic diodes that sustained their rectification performance at a stretch of λ = 3.0 [13]. Regarding biodegradation, Nyamayaro et al. incorporated cation- and anion-modified cellulose nanocrystals into an agarose hydrogel, achieving a high rectification ratio of up to 70, which surpassed that of conventional PSS/PDAC systems [44]. The device exhibited favorable biodegradability, offering a new avenue for the advancement of green chemistry.

The advantages of hydrogels mainly originate from their high ion mobility and facile chemical modification. By introducing oppositely charged polymer networks, p–n ionic junctions with high rectification ratios can be achieved. Meanwhile, the soft and hydrated nature of hydrogels provides excellent mechanical compliance and biocompatibility, making them attractive for wearable sensors, ionic skins, and biointegrated devices.

The core challenge for hydrogel materials lies in their environmental stability. Due to their high-water content, water evaporation causes gels to rapidly dehydrate, shrink, or even fracture in high-temperature (>50 °C) or low-humidity environments, leading to a decline in ionic conductivity and failure of rectification [45,46]. Below 0 °C, water freezing severely impedes ion transport [13,30]. Although introducing anti-freezing agents such as polyols (e.g., ethylene glycol and glycerol) [47,48] to form organo-hydrogel binary solvent systems can partially mitigate these issues, this approach concurrently compromises the ionic conductivity of the material.

### 2.2. Organogel

Organogels represent a crucial class of materials positioned between hydrogels and ionogels. Compared to hydrogels, organogels mitigate solvent evaporation and freezing issues through the integration of organic solvents [38,43]. Distinct from ionogels, they offer lower manufacturing costs and superior ionic conductivity, while allowing for property optimization by tuning solvent ratios [49]. In a notable study, Jiang et al. reported ionic diodes based on two organogel polymer electrolytes: PMMA/PC and PVDF-HFP/PC. These devices leverage the solubility differential of Zn^2+^ and Cl^−^ across different gels to establish an electric double layer, achieving rectification via asymmetric ionic diffusion and solubility. This configuration yields a rectification ratio of 23.1 and enables operation over a wide temperature range from −20 °C to 125 °C [49]. Ultimately, the core advantage of organogels in ionic diodes lies in their ability to simultaneously deliver high rectification ratios and robust wide-temperature stability.

The introduction of organic solvents improves environmental stability but usually decreases ionic conductivity because of increased solvent viscosity and reduced ion dissociation efficiency. Therefore, organogels typically exhibit an intermediate performance profile between hydrogels and ionogels, balancing ionic transport and environmental robustness.

### 2.3. Ionogel

Ionogels are semi-solid electrolytes formed by encapsulating ionic liquids within a polymer network, uniquely combining advantages such as high ionic conductivity and a wide electrochemical window [38,50,51]. Leveraging these traits, Khan et al. integrated an ionogel with monolayer MoS_2_ to achieve a self-bias effect through an asymmetric electrode coverage design [52]. Under forward bias, the ionogel narrows the Schottky barrier width to enhance electron injection, whereas under reverse bias, it depletes electrons within the channel, thereby yielding a rectification ratio of approximately 1000. Alternatively, Choi et al. employed a fibrous twisted architecture wherein TFSI^−^ anions within the ionogel induce electrochemical oxidative doping of P3HT under forward bias to enable current conduction, while de-doping under reverse bias suppresses the current, achieving a rectification ratio of around 100 (Figure 2c) [53]. These studies demonstrate that ionogels can realize solid-state ionic diodes with high rectification ratios by modulating interfacial barriers or driving electrochemical doping.

Nevertheless, the development of ionogels is accompanied by distinct challenges. First, restricted ionic diffusion within the polymer network inherently limits the response speed of the devices [49]. Second, many ionic liquids exhibit toxicity or poor biodegradability [54], which constrains their utilization in biomedical fields. Furthermore, the compatibility between the ionic liquid and the polymer network demands precise tailoring [55], as phase separation can inevitably lead to performance degradation.

Ionogels consist of ionic liquids confined within polymer networks, combining the high ionic stability of ionic liquids with the mechanical integrity of polymer matrices. The negligible vapor pressure of ionic liquids effectively suppresses solvent evaporation, enabling long-term operation over a wide temperature range.

### 2.4. Ionic Elastomer

Ionic elastomers are solid-state ionic conductors characterized by the immobilization of anionic or cationic salts onto polymer networks [56]. Unlike ionogels, they tether solid components within the polymer matrix; this solvent-free nature fundamentally eradicates issues related to solvent leakage and evaporation, positioning them as a rapidly emerging class of soft ionic conductors in recent years. In a pioneering study, Kim et al. developed a polyanion by anchoring sulfonate groups (-SO_3_^−^) and a polycation by securing quaternary ammonium groups (-N^+^(CH_3_)_3_) within separate poly (ethylene glycol) diacrylate networks. The resulting heterojunction established an electric double layer, yielding a rectification ratio of 50, which further escalated to 550 upon the integration of microporous carbon electrodes (Figure 2d) [31]. This device was subsequently configured into an ionic bipolar junction transistor, exhibiting an on/off ratio of approximately 40 while sustaining functionality under a tensile strain of λ = 1.6. Furthermore, under cyclic stretching from λ = 1.2 to 1.5, its ES/AT junction delivered an open-circuit voltage of 46 ± 2 mV and a power density of 1.6 nW/cm^2^, operating stably over 3500 cycles—offering a promising strategy for ionic elastomers in mechanical energy harvesting. Separately, Zheng et al. fabricated ionic elastomer diodes through a molecular chain oxygen-interpenetration strategy, achieving a shear strength of 317 kPa and a rectification ratio of 8.4, which remained robust after 200 bending cycles [57].

Their highly crosslinked or physically associated networks provide exceptional stretchability, toughness, and long-term stability, making them promising candidates for wearable and mechanically dynamic iontronic systems. However, the restricted segmental motion and limited free-ion concentration generally result in lower ionic conductivity compared with gel-based systems.

Despite these advantages, ionic elastomers suffer from relatively low ionic conductivity (10^−4^–10^−2^ S/m), which is 2 to 3 orders of magnitude lower than that of hydrogels, thereby impeding their implementation in high-frequency devices [58]. Additionally, the library of available ionic elastomer material systems remains somewhat narrow, necessitating further exploration and development [31].

## 3. Ionic Rectification Mechanisms

The rectification mechanism of soft ionic diodes is fundamentally different from that of traditional semiconductor p-n junctions. Specifically, ionic rectification mechanisms can be classified into four distinct categories. They exhibit distinct advantages and limitations in terms of ion transport behavior and material selection [38]. Polyelectrolyte p-n junctions generally provide high rectification ratios but suffer from slow response dynamics; discrepancies between ionic diffusion and migration mechanisms offer faster responses at the expense of weaker rectification [3,31]. Electrode reaction kinetics systems enable tunable rectification but are limited by long-term electrochemical stability, while Built-in asymmetric electric field systems provide structural scalability with moderate rectification performance [11,42].

Notably, an inherent trade-off exists between rectification ratio and response speed, as strong ion selectivity and pronounced depletion regions often hinder rapid ion transport. Therefore, the design of high-performance soft iontronic diodes requires balancing these competing factors according to specific application requirements. Table 2 provides a comparative analysis of the advantages and disadvantages of these four rectification mechanisms, offering readers a clearer understanding of their respective characteristics.

### 3.1. Polyelectrolyte p-n Junction Mechanisms

This represents the earliest proposed and most widely studied mechanism for ionic rectification [12,13,14,59,60,61]. In a polyanionic hydrogel (bearing fixed negative charges), mobile cations (e.g., Na^+^) serve as the majority carriers, which is analogous to a p-type semiconductor. Conversely, in a polycationic hydrogel (bearing fixed positive charges), mobile anions (e.g., Cl^−^) act as the majority carriers, mimicking an n-type semiconductor. Upon contact, counterions diffuse across the interface driven by the concentration gradient, establishing an ion depletion zone analogous to the depletion layer in a semiconductor p-n junction. Under forward bias (where the polyanion side is connected to the positive electrode and the polycation side to the negative electrode), counterions migrate toward the interface, narrowing the depletion zone and increasing the current. Under reverse bias, counterions are pulled away from the interface, widening the depletion zone and decreasing the current [33].

**Figure 3:**
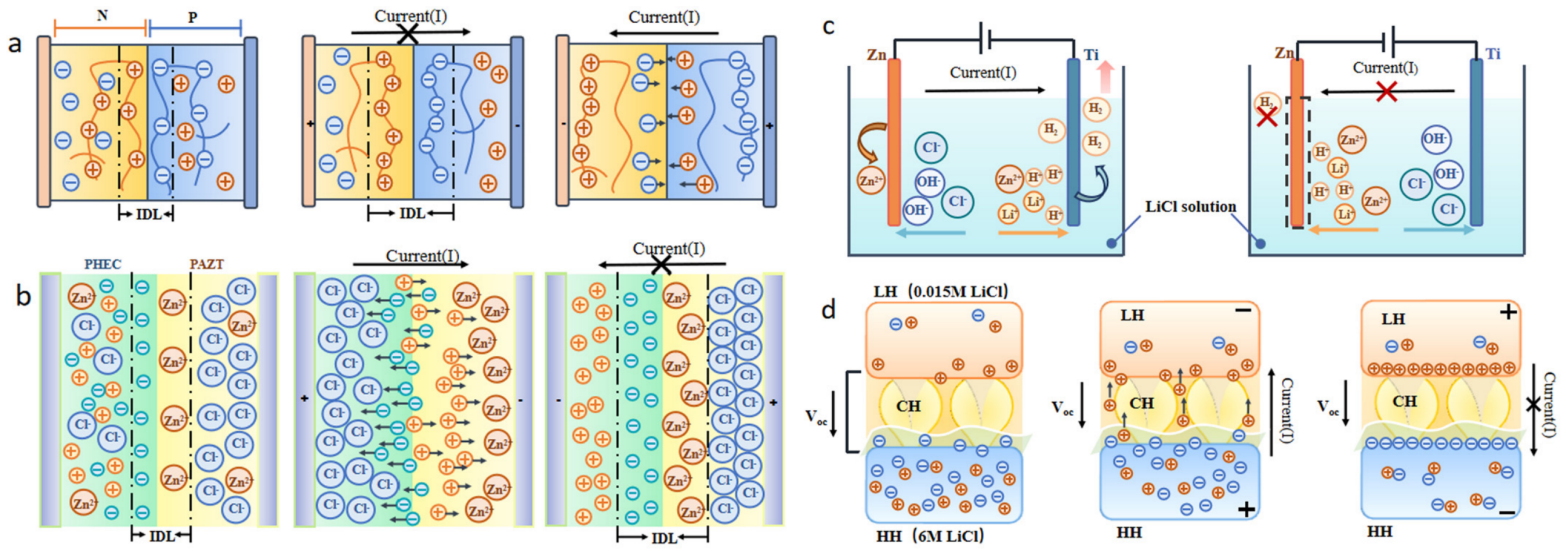
Schematics of four rectification mechanisms. (**a**) Polyelectrolyte p-n junction. (**b**) Discrepancy between ionic diffusion and migration. (**c**) Electrode reaction kinetics. (**d**) Built-in asymmetric electric field.

Kim et al. first systematically elucidated a similar rectification mechanism within an ionic elastomer system [31], which relies entirely on physical ion migration and electric double-layer (EDL) charging/discharging processes without any involved Faraday reactions (Figure 3a). When two ionic elastomers with oppositely fixed charges contact each other, mobile counterions form an interfacial ionic double layer (IDL). Under reverse bias, mobile ions are pulled away from the interface, thickening the IDL, which results in a low interfacial capacitance, high resistance, and blocked current. Under forward bias, mobile ions are driven toward the interface, disrupting the IDL and rendering the interface resistive, thereby allowing current to flow. The defining feature of this mechanism is that charge storage and release are mediated solely through the capacitive charging and discharging of the IDL, involving no cleavage or formation of chemical bonds. This mechanism offers distinct advantages, including the absence of side reactions, high device stability, rapid response times, and the ability to tune the rectification ratio by engineering the electrode capacitance. By utilizing microporous carbon electrodes to increase the EDL capacitance by an order of magnitude, Kim et al. [31]. successfully enhanced the rectification ratio from 50 to 550.

This mechanism is predominantly observed in hydrogel-based ionic diodes because hydrogels can easily incorporate fixed anionic or cationic groups, enabling the formation of ion-selective junctions and Donnan potentials. Although ionogels can also support this mechanism, the lower ion mobility of ionic liquids often leads to slower response dynamics.

### 3.2. Discrepancy Between Ionic Diffusion and Migration Mechanisms

Unlike the polyelectrolyte p-n junction that relies on fixed charges, the ionic diode reported by Jiang et al. is based on the differential ion diffusion and migration rates within two neutral gel electrolytes (Figure 3b). In the PAZT/PHEC system, Zn^2+^ is poorly soluble in PHEC, while Cl^−^ is poorly soluble in PAZT; consequently, these two ion species accumulate at the interface to form the IDL. In contrast, [EMIM]^+^ and CF_3_O_3_S^−^ exhibit high solubility in both gel polymer electrolytes (GPEs). Under forward bias, the highly mobile [EMIM]^+^ and CF_3_O_3_S^−^ ions cross the interface, shifting the device into a conducting state. Under reverse bias, the accumulation of Zn^2+^ and Cl^−^ is enhanced, which thickens the IDL and impedes ion transport [49]. The unique feature of this mechanism lies in its independence from fixed charges, achieving rectification solely by exploiting the differences in solubility and diffusion coefficients of different ions within distinct polymer matrices.

Rectification based on ion mobility mismatch can be implemented in various soft ionic materials but is particularly advantageous in organogel and ionogel systems, where solvent polarity and ion solvation environments can be tuned to modulate ionic transport coefficients.

### 3.3. Electrode Reaction Kinetics Mechanisms

The electrode reaction kinetics mechanism achieves ionic rectification by exploiting differences in electrochemical reaction rates across different electrode materials. The Zn/Ti hydrogel diode reported by Guo et al. exemplifies this unique category of rectification mechanism, which relies entirely on the vast disparity in hydrogen evolution overpotentials between the two electrodes (Figure 3c) [43]. The Zn electrode possesses a high hydrogen evolution overpotential, whereas the Ti electrode exhibits a low hydrogen evolution overpotential. Under forward bias, the hydrogen evolution reaction occurs on the Ti surface while an oxidation reaction takes place on the Zn surface, establishing a closed circuit that enables current conduction. Under reverse bias, H^+^ ions are attracted to the Zn surface; however, due to the high overpotential for hydrogen evolution on Zn, the reduction reaction is strongly suppressed, preventing circuit closure and resulting in a minimal current.

The key advantage of this mechanism is its exceptionally high rectification ratio, reaching up to 1201. Furthermore, the threshold voltage and operating characteristics can be tuned by selecting different electrode pairs. Additionally, with the introduction of an ethylene glycol/water binary solvent, the device can operate across a wide temperature window from −20 °C to 100 °C. The primary limitation of this mechanism is its strict dependence on specific electrode materials and the participation of H^+^, which restricts material selection and inherently involves the hydrolysis reaction of water.

Electrode-reaction-induced rectification has recently attracted increasing attention in ionogel and ionoelastomer systems because their stable interfaces and suppressed solvent evaporation facilitate reproducible interfacial electrochemical processes.

### 3.4. Built-In Asymmetric Electric Field Mechanisms

Lei et al. designed an asymmetric “trimer” hydrogel structure that utilizes a concentration gradient to construct a biomimetic built-in electric field for achieving ionic rectification (Figure 3d) [11]. This configuration consists of a high-concentration salt hydrogel layer (HH, 6 M LiCl), a low-concentration salt hydrogel layer (LH, 0.015 M LiCl), and an intermediate cation-selective layer (CH) bearing fixed negative charges. Driven by the concentration gradient between the HH and LH layers, cations tend to diffuse from the HH to the LH layer. Concurrently, the CH layer preferentially allows the passage of cations while repelling anions, leading to the spatial separation of positive and negative charges. This separation establishes opposing built-in electric fields at the HH/CH and CH/LH interfaces, generating an open-circuit voltage of approximately −180 mV. The paramount advantage of this mechanism lies in its ability to spontaneously establish a biomimetic resting potential relying solely on concentration gradients without requiring an external power source. Furthermore, it features a high degree of functional integration, enabling the simultaneous realization of rectification, synaptic plasticity, multimodal memory, and logic responses.

The rectification is largely independent of material chemistry and instead relies on asymmetric transport pathways and electric double-layer overlap. Consequently, this mechanism can be implemented in hydrogels, ionogels, and other soft ionic materials provided that suitable micro- or nano-structured architectures are available.

## 4. Applications

By virtue of their unique attributes—including ionic signal carriers, mechanical flexibility, and biocompatibility—soft ionic diodes hold immense promise for a wide range of application prospects across multiple frontier fields.

### 4.1. Logic Gating

**Figure 4:**
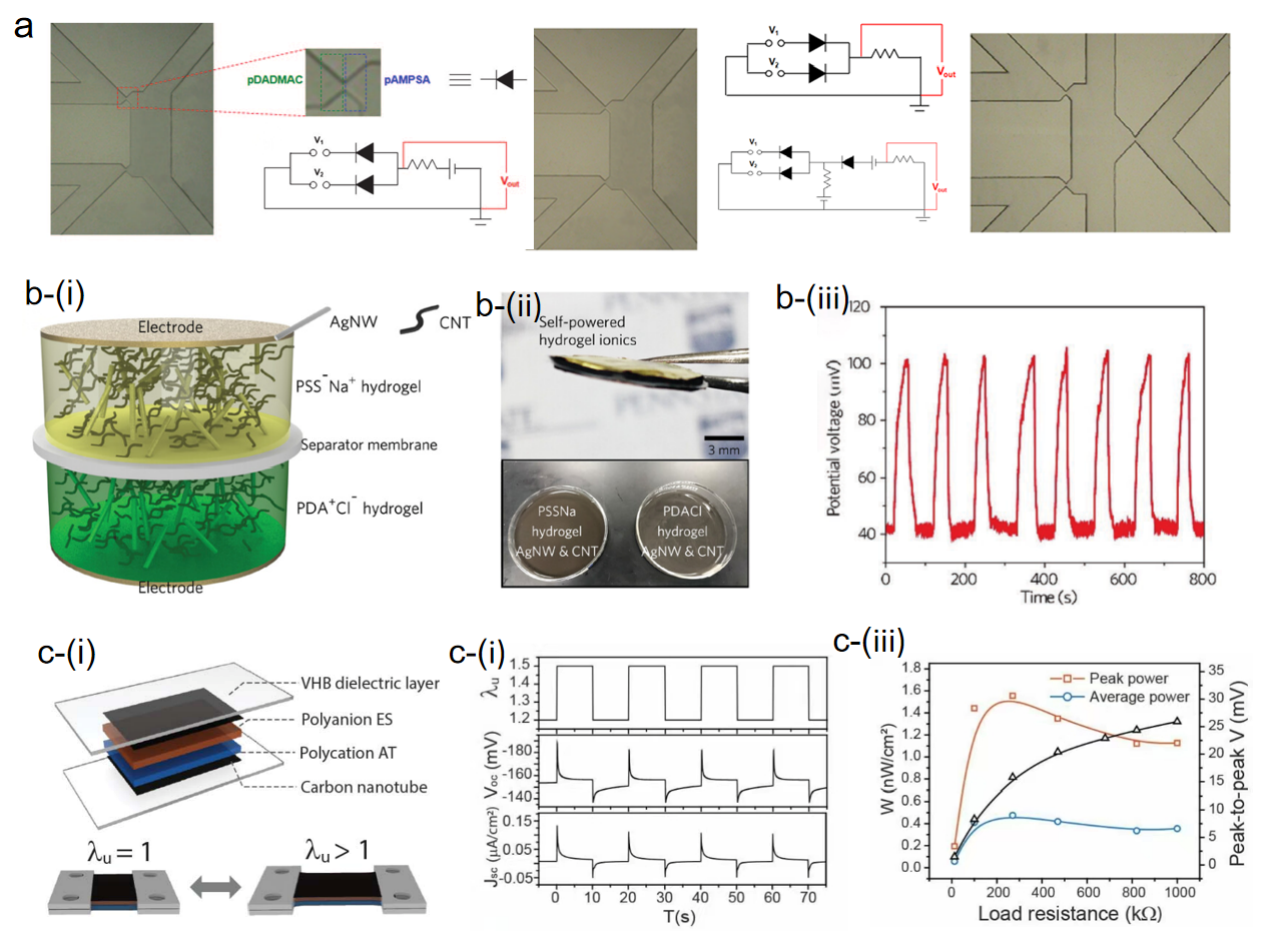
(**a**) The equivalent circuits of the AND, OR and NAND logic gate and their microchip pattern [67]. (**b-(i)**) Schematic illustration of hydrogel ionic diode. (**b-(ii)**) Photographs of self-powered hydrogel device (top) and molded hydrogel nanocomposites. (**b-(iii)**) Schematic illustration of energy-harvesting measurement with a forward connection for current density [70]. (**c-(i)**) Schematic illustration of an ES/AT electromechanical transducer. (**c-(ii)**) V_oc_ and J_sc_ under 0.05 Hz square-wave strain. (**c-(iii)**) Power output under 1 Hz sinusoidal stretching as a function of load resistance [31].

The interconnection of multiple ionic diodes can form ionic logic gates capable of executing fundamental Boolean operations [62,63,64,65,66]. Research indicates that the series or parallel configuration of two ionic diodes can construct AND or OR logic gates, whereas more complex logical functions, such as XOR and NAND, can be realized through the coupling of three or more devices. As early as 2009, Han et al. reported AND, OR, and NAND logic gates based on microchip polyelectrolyte diodes (Figure 4a) [67]. By utilizing fluorescent dyes to visualize ion distribution, they achieved dual-modal electrical and optical outputs, laying the foundation for the development of ionic integrated circuits. Furthermore, Wang et al. employed stretchable hydrogel ionic diodes to construct AND and OR gates, validating their logical functionalities even under stretched states [14]. This work demonstrates the profound potential of soft ionic logic devices for applications in wearable electronics.

### 4.2. Energy Conversion

In energy-related applications, ionic diodes primarily function in two key areas: mechanical energy harvesting and alternating current-to-direct current (AC-to-DC) rectification [68,69]. Zhang et al. reported a hydrogel ionic diode tailored for ultra-low-frequency mechanical energy harvesting. By embedding carbon nanotubes (CNTs) and silver (Ag) nanowires into a PSS/PDAC hydrogel p-n junction (Figure 4(b-(i)–b-(iii))) [70], mechanical compression disrupts the ionic equilibrium, driving ion migration to generate electrical current. Under an ultra-low frequency of 0.01 Hz, this device delivers an output voltage of 60 mV and a current density of 80 µA/cm^2^, which are several orders of magnitude higher than existing state-of-the-art technologies and perfectly match low-frequency human motions. Similarly, the ionic elastomer diode presented by Kim et al. generates an open-circuit voltage of 46 mV and a short-circuit current density of 0.18 µA/cm^2^ under square-wave stretching at 0.05 Hz (Figure 4(c-(i)–c-(iii)) [31], exhibiting stable performance over 3500 cycles. Although its power density is limited to 1.6 nW/cm^2^, its liquid-free nature renders it highly promising for long-term implantable energy harvesters. Furthermore, Guo et al. integrated a four-diode full-wave rectification circuit with a triboelectric nanogenerator (TENG) to convert the AC output of the TENG into DC for capacitor charging [43]; consequently, 4.7 µF and 10 µF capacitors were successfully charged to 1.7 V within 51 s and 127 s, respectively. This work represents a classic paradigm for the practical deployment of ionic diodes in energy harvesting systems.

Ionic diodes are also applicable in salinity gradient power generation, where nanofluidic diodes can efficiently harvest osmotic energy between seawater and freshwater to generate electricity. Zhang et al. investigated a polyelectrolyte hydrogel/aramid nanofiber heteromembrane for osmotic power generation. This heteromembrane induces an ionic diode effect by exploiting the asymmetries in structure, charge, and wettability between the two material layers, thereby achieving unidirectional ion transport. This effect successfully suppresses concentration polarization and mitigates the dissipation of Gibbs free energy as Joule heating. Meanwhile, the hydrogel layer provides a charged three-dimensional transport network that substantially enhances interfacial ion transmission efficiency. Experimental results demonstrate robust energy conversion performance that meets commercial benchmarks [71]. Consequently, polyelectrolyte hydrogels hold immense potential as high-performance interfacial materials for salinity gradient power generation. Among energy-related applications, self-powered biosensors driven by salinity or biochemical gradients may represent the most promising near-term direction, although substantial improvements in power density and long-term stability are still required for practical deployment [15,38].

### 4.3. Neuromorphic Information Processing

The ion migration kinetics of ionic diodes possess an intrinsic similarity to the neurotransmitter release-and-uptake process in biological synapses, garnering significant attention in the field of brain-like information processing. By constructing dielectric interfaces (e.g., polyanionic/polycationic hydrogel interfaces) or asymmetric structures, soft ionic conductor-based diodes achieve selective regulation of ion migration and rectification properties [32,33,72,73,74,75]. Under electrical pulse stimulation, these devices can mimic key signaling characteristics of biological synapses, including synaptic plasticity behaviors such as excitatory/inhibitory postsynaptic potentials, paired-pulse facilitation/depression, and long-term potentiation/depression [49,76,77,78,79]. Their operating mechanisms rely primarily on the enrichment and dissipation of ions at the interface, relaxation behavior driven by slow ion backflow, and variations in ion migration rates enabled by structural gradients or interfacial energy barriers, thereby endowing the devices with controllable conductance memory effects [80,81,82,83,84].

**Figure 5:**
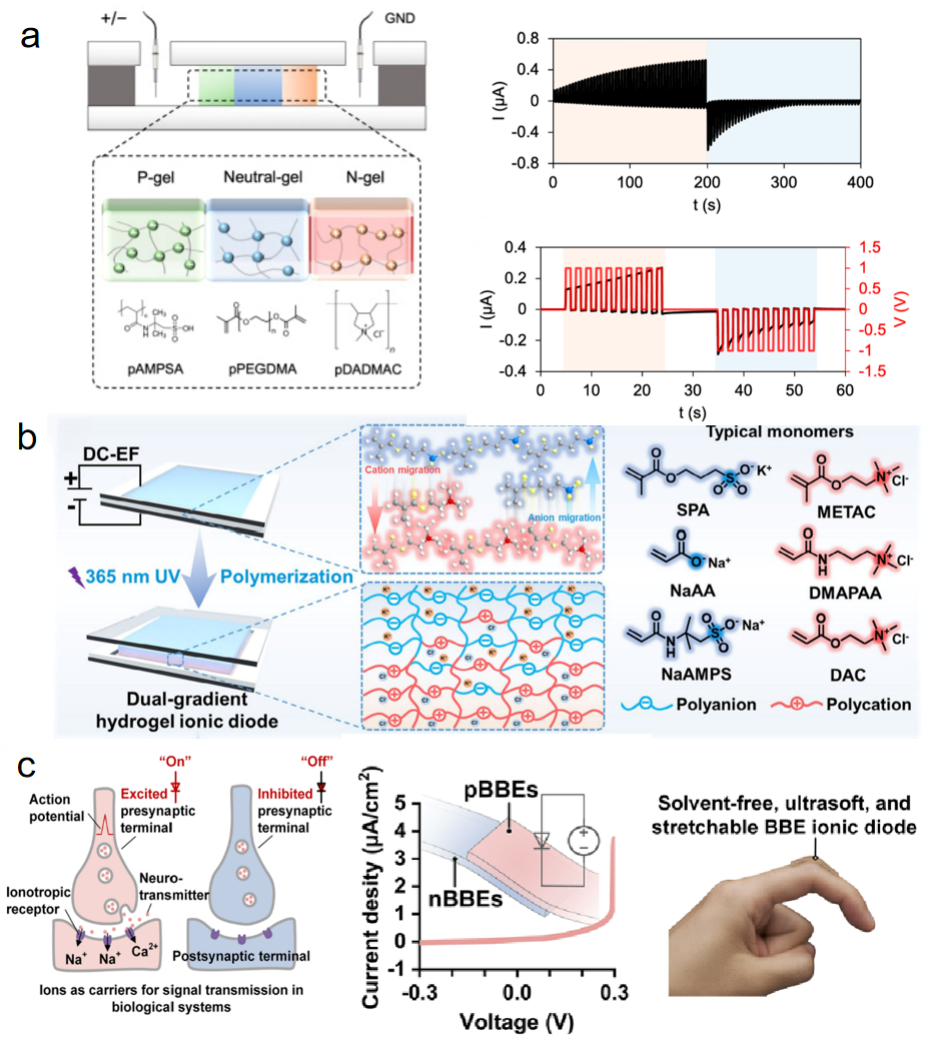
(**a**) Schematics of the iontronic bipolar memristor structure and operation and pulse signal processing [80]. (**b**) Fabrication of the dual-gradient hydrogel ionic diode [15]. (**c**) Schematic of directional ion flow in biological systems, BEE heterojunction and the photograph of the ultrasoft BEE diode [16].

Based on the aforementioned mechanisms, soft ionic diodes exhibit diverse application forms within neuromorphic systems. In the context of brain-like information processing, Zhang developed a three-layer bipolar polyelectrolyte gel memristor that effectively simulates critical functions of biological synapses (Figure 5a) [80]. By applying voltage pulse trains, the device demonstrates typical synaptic plasticity features and responds to continuous pulse sequences, achieving multi-level conductance state tuning; moreover, it enables non-destructive reading using low-frequency AC signals and exhibits excellent pulse processing capabilities. These characteristics allow the device to emulate ion signal-based information processing and learning processes in the human brain via ion migration. Concurrently, Han et al. constructed an all-aqueous, two-terminal ionic synaptic device utilizing a bipolar membrane ionic diode coupled with a chemical precipitation/dissolution process [76]. This work achieved the simultaneous realization of four fundamental synaptic plasticity behaviors—long-term potentiation, long-term depression, short-term potentiation, and short-term depression—on a single ionic platform for the first time. By integrating multiple excitatory and inhibitory ionic synapses, they successfully demonstrated dendritic signal integration functionality analogous to that found in hippocampal neural circuits. Furthermore, Lei and Wu designed an asymmetric “tri-unit” structured hydrogel ionic device that constructs a biomimetic resting potential and a built-in electric field by introducing a charged hydrogel layer between high- and low-concentration electrolyte hydrogels, thereby achieving precise spatiotemporal control over the ion flux distribution [11]. This device not only simulates short-term synaptic plasticity but also achieves multimodal storage of sensory, short-term, and long-term memory through training with electrical pulses of varied times and frequencies. It can even complete the encoding and memorization of letter and number images within a gel array while exhibiting good optical transparency, large-deformation stability, and basic logic gate functions. These findings showcase the immense potential of hydrogel ionic devices in synaptic plasticity, multimodal memory, signal integration, and logical responses, offering a novel platform and design paradigm for the development of brain-like computing systems and seamless human–machine interfaces fully based on ionic signaling.

### 4.4. Stretchable Sensing

Soft iontronic diodes are particularly attractive for stretchable sensing because ionic transport is inherently compatible with biological signaling processes. Their low operating voltages, soft mechanical properties, and ability to interface directly with ionic environments enable sensitive detection of biochemical signals while minimizing tissue irritation. The mechanical and chemical compatibility of soft ionic diodes with biological tissues gives them a natural advantage in the field of biosensing [16]. Xiong et al. utilized a hydrogel ionic diode for label-free monitoring of nucleic acid amplification. Negatively charged DNA electrostatically adsorbs onto the surface of the N-type hydrogel (a polycation), which neutralizes the fixed positive charges and decreases the rectification ratio [17]. The device can detect DNA concentrations below 1 ng/µL, successfully enabling PCR process monitoring and real-time, in situ MDA monitoring. Additionally, Du et al. developed a self-powered, multifunctional ionic skin based on ionic diodes [18]. Featuring a sandwich structure (ionogel electrode/P-type hydrogel/N-type hydrogel/ionogel electrode), it can simultaneously sense pressure, strain, temperature, salinity, and pH. This work reveals, for the first time, two distinct mechanoelectrical transduction mechanisms: thickness-dominated self-induced potential changes and contact area-dominated voltage loss variations. In the context of biomimetic prosthetics, dual-gradient hydrogel ionic diodes have been employed as ultrasensitive ionic skins to achieve self-powered tactile perception (Figure 5b) [15]. Exhibiting a high sensitivity of 1247.3 mV/MPa and an exceptionally low detection limit of 0.8 Pa, the skin can perceive contact with ultra-soft objects such as tofu and cream puffs. When integrated into smart prostheses, this ionic skin significantly enhances tactile sensing capabilities and control precision, enabling the damage-free manipulation of fragile objects. In soft robotics, stretchable ionic diodes serve as foundational components for fully soft logic control systems (Figure 5c) [16]. By integrating ionic diodes adjacent to the dielectric elastomers or pneumatic actuators of soft robots, researchers can achieve distributed, localized signal processing and actuation control, thereby reducing the reliance on rigid electronic components and bulky wiring.

## 5. Conclusions and Perspectives

Over the past decade, significant progress has been made in the field of soft ionic diodes. The material matrix has expanded from simple hydrogels to ionogels and ionic elastomers, enabling diverse performance combinations, ranging from high rectification ratios to broad operating temperatures, and from biodegradability to solvent-free operations. Concurrently, the underlying rectification mechanisms have evolved from classic polyelectrolyte p-n junctions to diverse principles, including asymmetric ion diffusion-migration and electrode reaction kinetics, thereby offering a rich suite of options for varied application scenarios. Beyond foundational current rectification, their application domains have extended into logic gating and energy conversion, demonstrating the immense potential of ionic diodes as a core component for next-generation flexible electronics.

The rectification ratio has been elevated from approximately 40 [12] reported by Cayre et al. to 1201 [43] by Guo et al. and 550 [31] by Kim et al. Concurrently, the operating temperature window has expanded from room temperature to a range spanning −20 °C to 125 °C [43,49], while the mechanical properties have advanced from brittle states [12] to stretchability exceeding 400% of the initial length [14], or even full elasticity. These breakthroughs establish a solid foundation for transitioning soft ionic diodes from laboratory-scale research to practical applications. Despite this rapid development, the field of ionic diodes remains in its infancy, demanding concerted efforts to address the following challenges:

(1) *Solvent volatilization and temperature range control:* The evaporation and freezing of water within hydrogel systems severely limit their long-term stability and environmental adaptability. Although ionogels circumvent the volatilization issue, the high cost of ionic liquids and their biocompatibility in specific systems remain critical concerns. Meanwhile, ionic elastomers eliminate solvent-related issues entirely, yet their low ionic conductivity remains a bottleneck that must be addressed.

(2) *Trade-off between ionic conductivity and mechanical performance:* High ionic conductivity typically necessitates a high solvent content or elevated ion concentrations, which inherently compromises the mechanical strength and elastic modulus of the material. Although strategies such as dual-network architectures and nanocomposite reinforcement can alleviate this trade-off to some extent, a fundamental resolution has yet to be achieved.

(3) *Trade-off between rectification ratio and response speed:* High rectification ratios often necessitate a thick depletion region or strong ion selectivity; however, this inherently increases the ion migration distance, thereby reducing the response speed. To date, no ionic diode has simultaneously achieved both a high rectification ratio and rapid response. This bottleneck is fundamentally limited by the intrinsically low mobility of ions, making it a physical barrier that is difficult to overcome in the near term.

(4) *Multi-ion crosstalk and device integration:* When integrating ionic circuits, different constituent devices may rely on distinct ionic species. Direct interconnected coupling can lead to cross-contamination of ions, the dissipation of concentration gradients, and subsequent performance drift [85]. Furthermore, the accumulation of parasitic resistance and the amplification of leakage currents scale with the number of cascaded logic gates, thereby severely restricting the integration scale. Currently, the experimental integration limit remains constrained to approximately a four-stage cascaded OR gate.

(5) *Lack of standardized performance metrics:* Discrepancies in voltage ranges, scan rates, electrolyte concentrations, and definitions of the rectification ratio across various studies hinder meaningful cross-study benchmarking [86]. Consequently, establishing a unified performance evaluation framework is an urgent imperative for the sustainable and healthy advancement of this field.

(6) *Integration density and scalability:* The feature sizes of soft ionic devices currently remain at the hundreds-of-micrometers to millimeter scale, lagging far behind the nanoscale dimensions of silicon-based transistors. This bottleneck stems not only from manufacturing process limitations but also from fundamental principles, as the diffusion coefficient governing ion migration dictates the minimum characteristic length scale. Exploring ion transport mechanisms that transcend the diffusion limit, such as confinement effects or the Grotthuss proton-hopping mechanism, may provide a crucial breakthrough [87].

From a long-term perspective, the evolution of soft iontronic diodes will unfold across three dimensions: material innovation, device architecture design, and paradigm shifts in computing.

(1) *Material dimension:* The development of novel ion conductors remains a continuous theme. Two-dimensional (2D) confined ion channels, such as graphene oxide and MXene membranes, hold the potential to enable ultra-fast ion transport, where their interlayer nanochannels can boost ion mobility by one to two orders of magnitude. Meanwhile, the tailorable pore structures of metal–organic frameworks (MOFs) and covalent organic frameworks (COFs) provide a molecular-level design space for ion selectivity. Additionally, natural biopolymers, including chitosan, sodium alginate, and cellulose, can further enhance biocompatibility and biodegradability, thereby meeting the stringent requirements of implantable and transient electronics. Although the application of MXenes, MOFs, and COFs in soft iontronic diodes remains very limited at present, these materials have already demonstrated promising ion transport and ion selectivity characteristics in nanofluidic and ion-selective membrane systems, suggesting considerable potential for future soft iontronic devices [47,68].

(2) *Device dimension:* Multifunctional integration represents a definitive trend; fusing sensing, rectification, energy conversion, and storage within a single soft iontronic device can drastically increase functional density per unit area. Furthermore, borrowing electronic design automation (EDA) tools from microelectronics to develop computer-aided design software tailored for soft iontronic devices will accelerate the development of complex ionic circuits.

(3) *System dimension:* If ionic transistors, diodes, and memristors can construct complete logic and memory units, information-processing systems utilizing ions as the sole carrier will become feasible. Such systems are inherently compatible with biological environments, require no signal conversion, and operate with ultra-low power consumption. Although this remains a long-term vision, preliminary breakthroughs in ionic logic gates, neuromorphic devices, and reservoir computing have already laid the foundation.

In summary, as a fundamental component of iontronics, the soft ionotronic diode is at a critical juncture, transitioning from proof-of-concept validation to practical application. Existing challenges such as dehydration and long-term stability, alongside the trade-offs between rectification ratio and response speed, provide continuous momentum and avenues for innovation in this field. Driven by the deep intersection of material science, electrochemistry, soft matter physics, and micro/nanofabrication, soft iontronics is poised to move beyond the laboratory into a broader spectrum of real-world applications.

Despite the promising potential of soft iontronic diodes for biomedical applications, biocompatibility remains a critical challenge, particularly for ionogel-based systems containing ionic liquids. Although ionic liquids possess negligible volatility and excellent ionic stability, their biological effects strongly depend on chemical structures, including cation/anion composition, alkyl chain length, and functional groups. Certain ionic liquids may induce cytotoxicity or inflammatory responses, especially under long-term exposure or leakage conditions. Therefore, the development of biocompatible ionic liquids and robust encapsulation strategies is essential for implantable applications.

From the perspective of technology readiness, wearable sensing and self-powered ionic skins appear to be among the closest application scenarios to practical deployment due to their relatively simple device architectures and direct compatibility with existing flexible electronics technologies [3,15]. In contrast, ionic computing and neuromorphic iontronic systems are still largely at the proof-of-concept stage and require substantial advances in integration density and device reproducibility [6,38].

## Figures and Tables

**Table 1:** Comparison of four materials.

Materials	Hydrogel	Organogel	Ionogel	Ionic Elastomer
Typical polymer matrix materials	PAAm, PVA	PVDF-HFP, PMMA	PS-PMMA-PS	ES/AT, PEGDA
Environmental stability	poor	good	good	excellent
Ionic conductivity (S·cm^−1^)	10^−3^–1	10^−4^–10^−2^	10^−5^–10^−2^	10^−5^–10^−4^
Stretchability (%)	200–2000	100–1000	50–1500	300–1550
Response speed	fast	medium	slow	medium
Maximum rectification ratio	1201	31	1000	550
Advantages	high conductivity	board temperature range	highly stable	leakage prevention
References	[12,13,14,38,39,40,41,42,43,44,45,46,47]	[42,48,49]	[50,51,52,53,54,55]	[31,56,57,58]

**Table 2:** Comparison of four ion current rectification mechanisms in ionic diodes.

Rectification Mechanisms	Polyelectrolyte p-n Junction	Discrepancy Between Ionic Diffusion and Migration	Electrode Reaction Kinetics	Built-in Asymmetric Electric Field
Salient features	fixed charges, Donnan exclusion	absence of fixed charges, solubility discrepancy	overpotential discrepancy	concentration gradient
Rectification ratio range	10–100	20–30	500–1200	5–10
Advantages	transparent mechanism, abundant materials	absence of fixed charges, board temperature adaptability	ultrahigh rectification ratio	self-powered, functional integration
Limitations	water-dependent, high prevalence of side reactions	limited rectification ratio	dependent on specific electrodes	limited ionic conductivity
Reference	[31]	[49]	[42]	[11]

## Data Availability

This review is based entirely on previously published studies. No new data were generated, collected, or analyzed, and no proprietary software or unpublished datasets were used. All data are available in the cited sources.
